# Serotonin, Kynurenine, and Indole Pathways of Tryptophan Metabolism in Humans in Health and Disease

**DOI:** 10.3390/nu18030507

**Published:** 2026-02-02

**Authors:** Milan Holeček

**Affiliations:** Department of Physiology, Faculty of Medicine in Hradec Králové, Charles University, Šimkova 870, 500 03 Hradec Králové, Czech Republic; holecek@lfhk.cuni.cz

**Keywords:** amino acids, melatonin, quinolinic acid, metabolic syndrome, metabolic disorders, neuroinflammation, cirrhosis, cancer, renal insufficiency

## Abstract

Tryptophan (TRP) is a proteinogenic and nutritionally essential amino acid involved in the formation of numerous bioactive substances. A crucial role in the TRP molecule is played by indole, a bicyclic ring formed by benzene and pyrrole, which confers hydrophobic and antioxidant properties and the ability to act as a ligand for aryl hydrocarbon and pregnane X receptors. The first parts of the article examine sources, nutritional requirements, and three pathways of TRP catabolism. Physiologically, ~5% of dietary TRP is catabolized through the pathway forming serotonin and melatonin in the brain and enterochromaffin cells of the gut, ~85% through the pathway resulting in the formation of nicotinamide nucleotides and kynurenine and its derivatives in the liver and immune cells, and ~10% in gut microbiota to indole derivatives. Alterations of individual TRP catabolism pathways in aging, alcoholism, inflammatory bowel disease, metabolic syndrome, renal insufficiency, liver cirrhosis, cancer, and nervous diseases, e.g., depression, Alzheimer’s and Parkinson’s diseases, multiple sclerosis, and schizophrenia, are examined in the central section. The final sections are devoted to the benefits and adverse effects of TRP supplementation, the therapeutic use of various TRP metabolites, and the pharmacological targeting of enzymes, transporters, and receptors involved in TRP catabolism. It is concluded that all pathways of TRP catabolism are altered across a broad spectrum of human illnesses, and further investigation is needed to understand their role in disease pathogenesis better. The goal for clinical research is to explore options for TRP-targeted therapies and their integration into new therapeutic strategies.

## 1. Introduction

Tryptophan (2-amino-3-(1*H*-indol-3-yl)propanoic acid) exists in two isoforms, L and D. The article is focused on L-isoform (Trp or W), a proteinogenic, both glucogenic and ketogenic, and for humans nutritionally essential amino acid, hereinafter referred to as TRP. The significance of D-tryptophan, a compound produced by bacteria that is not metabolized by humans but can act as a food preservative, a probiotic, and a non-nutritional sweetener, has recently been reviewed by Wang et al. [1].

Unlike other essential amino acids involved in protein synthesis, TRP metabolism through three pathways referred to as TRP-serotonin (TRP-SER), TRP-kynurenine (TRP-KYN), and TRP-indole (TRP-IND) results in the production of numerous bioactive substances. Fairly well-known are neurotransmitter serotonin and hormone melatonin, which have been used to treat depression and sleep disorders for many years. However, the roles of most other metabolites, the significance of the TRP-KYN and TRP-IND pathways, and the integration of TRP metabolism into a complex network of homeostatic metabolic reactions and disease-specific pathways, remain poorly understood. For example, not fully clarified are functions of pleiotropic compounds formed through TRP-KYN pathway, such as kynurenine (KYN), anthranilic acid (ANA), kynurenic acid (KYNA), and quinolinic acid (QA), capable modulate brain and immune system, and which play a role in the pathogenesis of a wide range of illnesses, including cancer, liver cirrhosis, and neurodegenerative and psychiatric diseases, such as dementia, Alzheimer’s disease, Parkinson’s disease, multiple sclerosis, and schizophrenia [2,3]. Recent studies have focused on the role of TRP metabolites formed by the TRP-IND pathway in the large intestinal microbiota for bowel function and host health, as components of pathways referred to, for example, as the gut–brain, gut–renal, and gut–liver axes [4,5,6].

**Figure 1:**
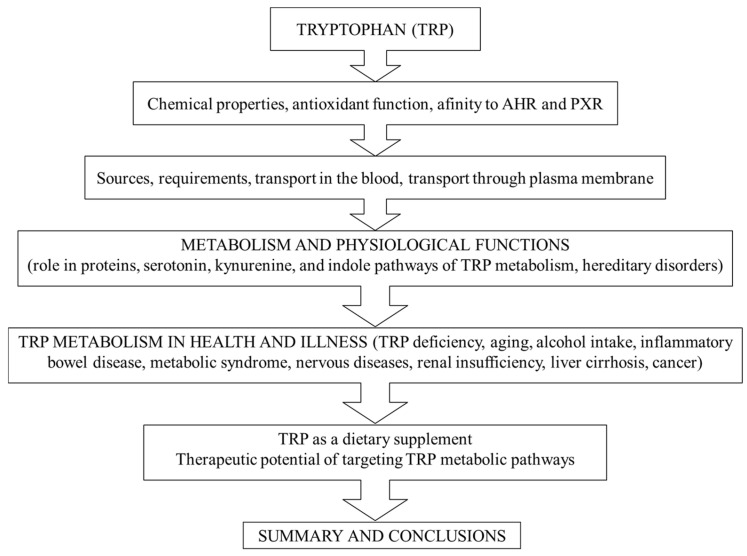
The conceptual framework of the article. AHR, aryl hydrocarbon receptor; PXR, pregnane X receptor.

This article aims to provide a comprehensive review of the role of TRP and its metabolites in healthy individuals and various physiological and pathological conditions, as well as the use of TRP as a dietary supplement, and the potential for targeting TRP metabolism pathways in the therapy of various diseases. The review will help reflect on what TRP supplementation and targeting its metabolic pathway can and cannot achieve, and encourage researchers to conduct studies that push this clinically important field of amino acid metabolism forward on a solid basis. Articles providing details on the specific topic for readers seeking more comprehensive sources of information are referenced. The article’s conceptual framework is shown in Figure 1.

## 2. Biochemical Properties of Tryptophan

The molecular weight of 204.22 g/mol and pI value of ~5.9 indicate that TRP belongs among large neutral amino acids (LNAAs), including valine, leucine, isoleucine, phenylalanine, tyrosine, and histidine. An important role in the TRP molecule is played by indole, a bicyclic ring formed by benzene and pyrrole:


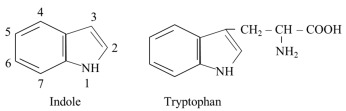


Due to the presence of the indole ring, TRP is degraded at high temperatures and during acid hydrolysis, which decreases its content during food processing and limits the accuracy of determining both free and protein-bound TRP concentrations using conventional methods [7]. Indole is also responsible for TRP hydrophobic features. Therefore, most TRP is transported in the blood bound to proteins, and some TRP metabolites diffuse across the plasma membrane, including the intestinal epithelium and the blood–brain barrier (BBB). The hydrophobic feature of TRP residues also plays a role in protein structure (Section 5). The indole ring is also responsible for the antioxidant properties of some TRP metabolites and the ability of TRP and its derivatives to act as ligands to aryl hydrocarbon receptors (AHRs) and pregnane X receptors (PXRs).

### 2.1. TRP Sensitivity to Oxidative Stress and Antioxidative Properties of TRP

The indole moiety is sensitive to the influence of a variety of reactive oxygen (ROS) and nitrogen (RNS) species, including hydrogen peroxide, hydroxyl radical, singlet oxygen, nitric oxide, and peroxynitrite anion. Multiple reaction pathways appear to occur in the reaction of ROS and RNS with the indole moiety, and multiple products have been identified, including 3-hydroxytryptophan, dioxindolylalanine, KYN, *N*-formylkynurenine, and dihydroxypyrroloindole [8]. Because the indole part of TRP can be degraded into multiple toxic products by exposure to light, high or low pH, heat, and oxygen, the storage of TRP-containing supplements, especially in solution form, must be under strict control [8]. A good target of oxidative stress is especially the TRP residues in proteins (Section 5). On the other hand, some TRP metabolites, such as KYNA and melatonin, are recognized as potent antioxidants (Section 6.3.2 and Section 7.2.3).

### 2.2. Anti-Inflammatory Properties of TRP

Anti-inflammatory properties of TRP and many of its derivatives are mediated by the antioxidant properties of the indole ring and affinity to AHR (Section 2.3). Using an in vitro cell-based assay, TRP isolated from human milk exhibited nearly 99-fold higher oxygen radical absorption capacity associated with a decreased response to endotoxin-induced formation of proinflammatory cytokines [9]. In this context, it should be noted that the anti-inflammatory effects of TRP and its derivatives could have both beneficial, e.g., attenuation of inflammation, and detrimental, e.g., weakening the ability to destroy pathogens and tumor cells, effects.

### 2.3. TRP and Aryl Hydrocarbon Receptor (AHR)

AHR is a transcription factor that resides in the cytosol as an inactive complex with chaperones, primarily in barrier tissues such as the skin, gut, and lungs, as well as in immune cells. The key AHR ligands include several exogenous and endogenous substances with an aryl chemical structure, such as flavonoids, dibenzofurans, benzopyrene, tetrapyrroles, and arachidonic acid metabolites. TRP, and some TRP metabolites, including indole, KYN, KYNA, indole-3-propionic acid (IPA), indole-3-acetic acid (IAA), skatole, and tryptamine, act as agonists of AHR [10,11,12,13]. Upon ligand binding, the AHR is translocated into the nucleus and induces transcription of target genes, primarily those encoding enzymes of the cytochrome P450 system (Cyp1a1, Cyp1a2, and Cyp1b1), the AHR repressor, and genes involved in apoptosis, cell proliferation, and differentiation. Hence, AHR plays a remarkable role in immune function, e.g., T-cell differentiation and cytokine formation, maintenance of the mucosal surface in the gut, and carcinogenesis [10,11].

The consequences of AHR activation depend on the specific ligands and cell types involved in the response. In the gut, AHR activation is involved in the detoxification of xenobiotic compounds and promotes signals, such as interleukin-22 expression, that help maintain mucosal homeostasis, antimicrobial defense, and gut barrier integrity [4,10].

### 2.4. TRP and Pregnane X Receptor (PXR)

PXR, also referred to as steroid or xenobiotic sensing receptor, is a nuclear receptor that acts as a transcription factor in response to a wide range of hydrophobic substances, such as steroids, bile acids, toxins, some drugs, and indoles (e.g., IPA, indole, and indole-3-acetamide). PXR is primarily expressed in the liver, intestine, and, to a lesser extent, in the kidney. Its activation induces the expression of genes involved in the elimination of xenobiotics from the body, such as those encoding cytochrome P450 and the MDR1 transporter. PXR activation also plays a role in the metabolism of glucose, ketone bodies, and fatty acids and suppresses NF-κB activity, a key regulator of inflammation and the immune response [14,15].

## 3. Sources, Requirements, and Transport of Tryptophan in the Blood and Through the Plasma Membrane

### 3.1. Sources of TRP

TRP is a nutritionally essential amino acid and, therefore, must be obtained in sufficient quantities from the diet. TRP content in most food proteins is 2–4 times lower when compared with the content of other nutritionally essential amino acids. Nutrients with relatively high content are meat, eggs, fish, and dairy products, such as chocolate. Potatoes and legumes are suitable vegetable sources. The TRP is completely lacking in gelatin and remarkably low in corn unless bred or genetically modified varieties [16,17].

The distribution of TRP in food proteins differs according to the specific protein fraction. Practically important is the high TRP content in human milk proteins, which include 28% α-lactalbumin, containing around 5.8% TRP. In contrast, lactalbumin in cow’s milk contributes to only 3% of total protein [18]. Hence, breast milk is a much better source of TRP when compared with cow’s milk, and it seems that TRP derived from breast milk is crucial for infants.

Gut microbiota capable of synthesizing and releasing TRP include *Escherichia coli* and *Corynebacterium glutamatum* [19]. Their contribution as a source of TRP for the host is small, if any, because most TRP is utilized, together with TRP reaching the colon from the upper parts of the gastrointestinal tract, for bacterial protein synthesis or catabolized in TRP-IND pathways (Section 4).

### 3.2. Nutritional Requirements

The overall dietary TRP requirements are the lowest among all nutritionally essential amino acids [20]. The estimated average TRP requirements and the recommended dietary allowance for adults, as suggested by the World Health Organization, are 4 mg/kg/day and 5 mg/kg/day, respectively [21]. Dietary TRP requirements are higher in subjects consuming vitamin B_3_-deficient diets to prevent pellagra (Section 9.1) and during pregnancy and in infants, ~10 mg/kg/day [21]. It has been shown that low TRP levels are associated with increased prevalence of depressive symptoms in pregnant women [22,23]. High TRP content in human milk plays a remarkable role in milk antioxidant potential and mitigates the formation of proinflammatory cytokines [9]. TRP levels in newborns are 2–4 times higher than in adults [24]. Hence, TRP intake influences maternal and fetal health outcomes.

### 3.3. TRP Transport in the Blood

The plasma TRP concentration in healthy people ranges from 40 to 60 µmol/L. Unlike other amino acids in plasma, the majority (80–90%) of TRP is bound to proteins, primarily albumin [25,26]. Several substances, such as fatty acids and TRP metabolites, can displace protein-bound TRP, thereby increasing free TRP levels [27]. Therefore, free TRP increases during stress and exercise due to lipomobilization and in patients with uremia due to increased levels of some TRP metabolites. In addition, it has been shown that valproic acid (a drug used to treat epilepsy) can displace protein-bound TRP, increase free TRP levels, and affect serotonin synthesis in the brain [28]. Increased free and decreased concentrations of total TRP can be observed in patients with liver cirrhosis due to hypoalbuminemia [26,29].

### 3.4. TRP Transport Through the Plasma Membrane

Transport of TRP across the plasma membrane is ensured by several systems for neutral amino acids, primarily the B^0^ system (B^0^AT1, SLC6A19), L-systems, including LAT1 (SLC7A5) and LAT2 (SLC7A8), and the T system (TAT1, SLC16A10). B^0^AT1 is a Na^+^-dependent transporter responsible for TRP resorption through the apical membrane of enterocytes of the small intestine and proximal tubules of the kidneys. L-systems LAT1 and LAT2 are ubiquitous heteromeric transporters that allow the antiport of TRP and other LNAA by facilitated diffusion. Transporter T (TAT1) mediates the unidirectional transport of aromatic amino acids (AAA; Phe, Tyr, and TRP) from enterocytes and proximal tubular cells into the blood [20].

Because of the competitive transport among the individual LNAA through B^0^ and L systems, the absorption of TRP in the gut and its removal from circulation are influenced by concentrations of other LNAA. Because dietary proteins contain less TRP than other LNAA, and BCAA are, unlike TRP, not catabolized in the liver, ingestion of a protein-rich meal decreases the TRP to LNAA ratio in the blood, which may decrease TRP transport through the L system into the brain. Due to increased BCAA levels, the TRP-to-BCAA ratio also decreases in starvation, insulin resistance, and diabetes [30,31]. On the other hand, insulin released after carbohydrate-rich meals increases the TRP to LNAA ratio, as BCAA is preferentially used for protein synthesis [32]. Clinically significant is also the decrease in BCAA levels in hyperammonemia, leading to an increased AAA-to-BCAA ratio, which plays a role in the pathogenesis of hepatic encephalopathy [29,33,34,35,36].

#### Hereditary Disorders of TRP Transport Through the Plasma Membrane

- *Hartnup’s disease*—a disorder of transport of TRP and other LNAA in the proximal tubules of the kidney and small intestine due to a mutation in SLC6A19 (B^0^AT1). It is clinically manifested by aminoaciduria and symptoms of pellagra, which respond to therapy with niacin, but not to TRP administration [17].
- *Drummond’s (blue diaper) syndrome*—a rare disease caused by a disorder of TRP resorption in the small intestine due to TAT1 (SLC16A10) mutation. The result is increased TRP degradation by the intestinal microbiota into indole and excretion of indican in the urine [37].

## 4. The Pathways of Tryptophan Metabolism

Assuming the TRP content in most proteins is 1–2% [7] and protein intake of an adult man of body weight 70 kg is 70 g per day, the normal daily TRP intake in food is 0.7–1.4 g, i.e., 10–20 mg/kg of body weight. The main routes of TRP metabolism in humans include synthesis of proteins and degradation through the kynurenine pathway (TRP-KYN pathway) in the liver and immune cells, the serotonin pathway (TRP-SER pathway) in the small intestine and the brain, and indole pathways leading to the formation of indole and its derivatives (TRP-IND pathways) by microbiota in the large intestine.

With the TRP content in most body proteins between 1–2% [7] and the protein turnover in an adult of ~300 g per day [38], 3–6 g of TRP is used under normal conditions daily for protein synthesis and released during protein breakdown. Articles examining TRP metabolism have reported that at least 95% of ingested TRP is degraded via the TRP-KYN pathway, and less than 5% via the TRP-SER pathway [17,24,39,40]. However, these reports neglect the microbiota-mediated metabolism of dietary TRP in the colon.

Important insights into the role of the microbiota in dietary TRP metabolism have been provided by early studies demonstrating that approximately 12 g of free amino acids and proteins per day reach the large intestine. More than half is composed of food proteins, while the rest consists of digestive juices, mucus, and desquamated enterocytes [41]. In the large intestine, proteins are broken down into amino acids by proteases derived from the upper parts of the digestive system and by bacteria. Assuming that 6 g of proteins appearing in the large intestine is from the diet, and the average amount of TRP in proteins is 1.5% [7], approximately 0.10 g (~10%) of TRP of dietary origin is offered to gut microbiota to be used for the synthesis of microbial proteins or catabolized.

**Figure 2:**
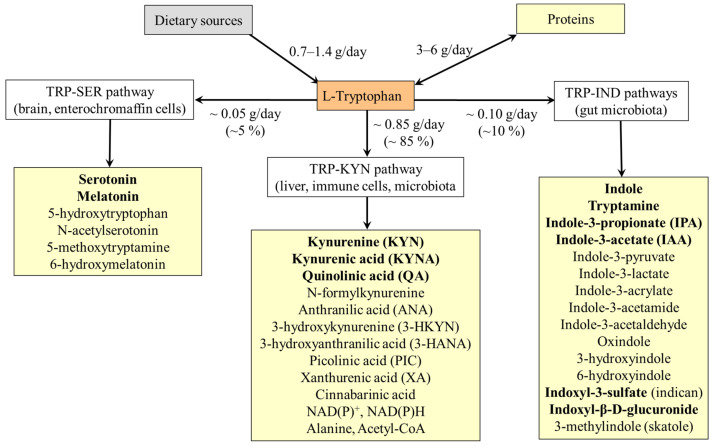
The pathways and main products of TRP metabolism in man. Metabolites mentioned in more detail in the article are highlighted in bold.

In summary, because, under usual conditions, there is a balance between TRP use for protein synthesis and its release from protein breakdown, approximately 5% (0.05 g) of TRP of food origin is metabolized through the TRP-SER pathway, 85% (0.85 g) through the TRP-KYN pathway, and 10% (0.10 g) is available for microbiota in the large intestine (Figure 2).

## 5. Tryptophan and Proteins

The TRP content in proteins is lower than that of other proteinogenic amino acids [7]. Higher TRP contents are associated with α-lactalbumin in human milk and acute-phase proteins, such as C-reactive protein, haptoglobin, and fibrinogen, which are synthesized by the liver in response to inflammatory challenges [42,43]. In membrane proteins, TRP plays a role in the stability and orientation of transmembrane proteins, serving as a membrane anchor for proteins residing near the lipid-water interface [44]. TRP residues in proteins, especially the pyrrole ring of the indole nucleus, are susceptible to oxidation by ROS and can contribute to altered structure, protein function, and the pathogenesis of various disorders [45]. In humans, oxidized TRP residues have been identified in apolipoprotein A1 (apoA1) recovered from human atheroma [46].

## 6. TRP-SER Pathway and Physiologic Role of Serotonin and Melatonin

### 6.1. TRP-SER Pathway

**Figure 3:**
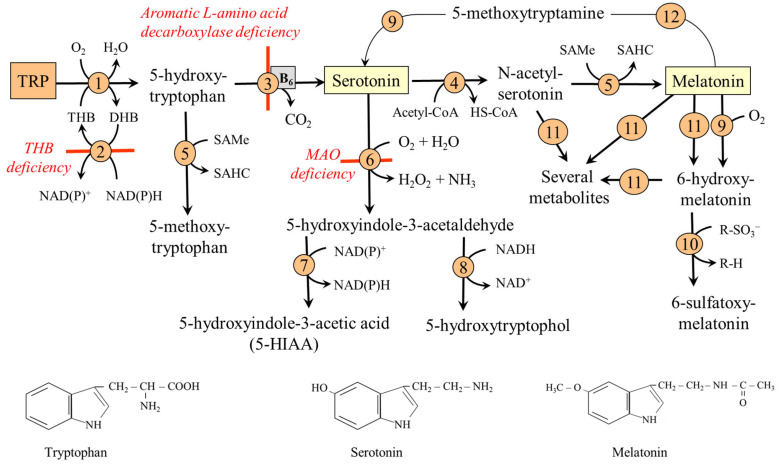
The TRP-SER pathway and routes of serotonin and melatonin catabolism. 1, TRP hydroxylase; 2, dihydrobiopterin reductase; 3, aromatic L-amino acid decarboxylase; 4, serotonin-*N*-acetyltransferase; 5, hydroxyindole-*O*-methyltransferase (*N*-acetylserotonin-*O*-methyltransferase); 6, monoamine oxidase; 7, aldehyde dehydrogenase; 8, aldehyde reductase; 9, cytochrome P450 enzymes; 10, sulfotransferase; 11, ROS and oxidases; 12, melatonin deacetylase. DHB, dihydrobiopterin; THB, tetrahydrobiopterin. SAMe, S-adenosylmethionine; SAHC, S-adenosylhomocysteine.

The main products of the TRP-SER pathway are serotonin and melatonin (Figure 3). The first and rate-limiting enzyme of the pathway is TRP hydroxylase (TRPH), which exists in two isoforms, TRPH1 and TRPH2. TRPH1 is highly expressed in the gut in enterochromaffin cells (ECCs) and in the pineal gland. TRPH2 expression in the brain is restricted to serotonergic neurons in the raphe nuclei of the reticular formation in the brain stem. Outside the brain, the TRPH2 is found in enteric serotonergic nerves [47]. Nakamura et al. [48] have demonstrated that the K_m_ of TRPH1 for TRP depends on tetrahydrobiopterin (THB) concentration and ranges between 7.5 and 16.6 µM. In contrast, the K_m_ of TRPH2 does not depend on THB level and is ~19.2 µM. The data suggest that serotonin synthesis is proportional to changes in TRP availability and TRPH1 synthesizes serotonin more efficiently than TRPH2 [48]. 5-hydroxytryptophan, produced by TRPH1/2, is a direct substrate for serotonin synthesis.

Serotonin is synthesized from 5-hydroxytryptophan by aromatic L-amino acid decarboxylase, which catalyzes the decarboxylation of L-DOPA and 5-hydroxytryptophan to dopamine and serotonin, respectively. Most (~90%) is formed in the ECC of the small intestine, while approximately 10% is formed in the raphe nuclei of the brain stem and the pineal gland. Small, but clinically important amounts are synthesized in epithelial, neuroendocrine, and mesenchymal cells, such as fibroblasts, in the lung, pancreas, adipose tissue, and vascular wall, where TRPH1 expression has been proven [49,50,51,52].

Most of melatonin (5-methoxy-*N*-acetyltryptamine) is synthesized from serotonin by serotonin-*N*-acetyl transferase and hydroxyindole-*O*-methyltransferase in the pineal gland; extra pineal sources include retina, bone marrow, platelets, skin, and ECC. Additional sources of melatonin for humans include gut microbiota and the diet, e.g., milk [53].

#### 6.1.1. Serotonin Degradation

Serotonin degradation is initiated by the action of monoamine oxidase (MAO) to form 5-hydroxyindole-3-acetylaldehyde. There are two types of MAO, MAO-A and MAO-B, found in presynaptic parts of neurons, which form serotonin, dopamine, or norepinephrine. Type A is also found in the liver, lungs, and enterocytes, while type B is primarily located in blood platelets [54,55]. Under usual conditions, most of the 5-hydroxyindole-3-acetylaldehyde formed by MAO is oxidized to 5-hydroxyindole-3-acetic acid (5-HIAA), which is released in urine; much less is reduced to 5-hydroxytryptophol [56]. The second possibility is promoted by acute alcohol intake due to the surplus of NADH formed during ethanol conversion to acetate (ethanol + NAD^+^ → acetaldehyde + NADH).

#### 6.1.2. Melatonin Degradation

The primary metabolic pathway of melatonin degradation involves hydroxylation by hepatic cytochrome P450 enzymes to form 6-hydroxymelatonin, which is subsequently conjugated with sulfate and excreted in the urine. Measuring the amount of 6-sulfatoxymelatonin excretion is a reliable method for evaluating the total amount of melatonin produced [57]. Various other metabolites formed from melatonin by interaction with ROS and RNS possess biological and pharmacological properties [58]. Under in vitro conditions, it was demonstrated that melatonin can be deacetylated to 5-methoxytryptamine, a substrate for cytochrome P450 enzymes to produce serotonin. Hence, a cycle of serotonin → *N*-acetylserotonin → melatonin → 5-methoxytryptamine → serotonin can play a role in serotonin and melatonin homeostasis [59].

#### 6.1.3. Hereditary Disorders of the TRP-SER Pathway

- *Tetrahydrobiopterin (THB) deficiency*. THB is required as a cofactor of phenylalanine hydroxylase, tyrosine hydroxylase, and TRPH. Defects in the biosynthesis of THB lead to deficiencies of dopamine and serotonin in the central nervous system. The most common cause is a deficiency of dihydrobiopterin (DHB) reductase, which is required to convert DHB back into THB. The symptoms include low muscle tone, movement disorders, impaired thermoregulation, and neurological, behavioral, and developmental problems. Treatment consists of THB supplementation and replacement therapy with catecholamines (L-DOPA) and serotonin precursors [60].
- *Aromatic L-amino acid decarboxylase deficiency*. A rare autosomal recessive disorder leading to a combined deficiency of dopamine, norepinephrine, epinephrine, and serotonin. The main clinical symptoms, which typically emerge in the first months of life, include hypotonia, hypokinesis, autonomic dysfunction, and developmental delay [61].
- *MAO-A deficiency*. MAO-A deficiency (Brunner syndrome) is a rare disorder characterized by elevated levels of monoamines, such as serotonin, dopamine, and norepinephrine in the brain, and reduced urinary levels of 5-HIAA and vanillylmandelic acid. Symptoms include intellectual disability, obsessive behavior, and episodic explosive aggression, flushing, headaches, and diarrhea [56].

### 6.2. The Role of Serotonin

The effects of serotonin are mediated through seven families of serotonin (5-hydroxytryptamine, 5-HT) receptors expressed throughout the body. Six of them (5-HT_1–2_ and 5-HT_4–7_) act as G-protein coupled receptors modulating adenylyl cyclase or phospholipase C signal transduction pathways; the 5-HT_3_ receptor acts as a ligand-gated ion channel [49]. In some cells, such as neurons, thrombocytes, and mast cells, serotonin transporters facilitate the uptake of serotonin from the extracellular space. In recent years, the focus of attention has been on serotonylation, the attachment of serotonin to intracellular proteins, which plays a role in hemostasis, smooth muscle contraction, neuronal differentiation, insulin secretion, and epigenetic regulation of gene expression, including cell proliferation and apoptosis [62]. Because serotonin cannot cross the BBB, the effects of serotonin produced in the brain and in the periphery are separated.

#### 6.2.1. Serotonin and the Brain

Serotonin, formed through TRPH2 in the raphe nuclei of the brain stem, is delivered via axons to various brain areas. Because the enzyme is ~50% saturated with its TRP substrate, alterations in TRP availability influence brain serotonin formation [48,63]. Using pharmacological manipulations, such as the administration of serotonin reuptake inhibitors and TRP-free amino acid solutions, it has been demonstrated that serotonin induces a feeling of satiety, decreases pain sensitivity, inhibits aggressive behavior, and modulates feelings of fatigue, sexual behavior, learning, and memory [64]. In addition, serotonergic neurons respond to cold and, through activation of the sympathetic nervous system, increase metabolic turnover and energy expenditure in brown adipose tissue [65,66].

#### 6.2.2. Serotonin and the Gut

More than 90% of serotonin is formed in the gut by ECC through TRPH1, which is activated by various signals, including gut contractions, ingested nutrients, and gut microbiota metabolites, such as short-chain fatty acids and indole and its metabolites [67]. In the gut, the primary effects of serotonin include stimulation of intrinsic reflexes, such as segmentation contractions, the secretion of digestive juices and enzymes, and the proliferation of mucosal cells. Via modulation of the activity of afferent fibres of the parasympathetic vagal nerve, it influences the gut–brain axis and the feeling of satiety, pain, and nausea [68]. Small amounts of serotonin synthesized by neurons of the enteric nervous system through TRPH2 are involved in peristalsis and promote the proliferation of mucosal cells and neural regeneration [47].

#### 6.2.3. Other Serotonin Effects

Most serotonin of ECC origin is released into the circulation, exerting an apparent influence on the body’s metabolism. Serotonin presence in the blood is responsible for the vasoconstrictive properties of serum from which it has been isolated, hence its name [69]. Most of the serotonin released by ECC into the blood is collected by thrombocytes and stored in delta granules. Its release from activated platelets during the so-called release reaction is essential for hemostasis. Furthermore, serotonin of ECC origin plays, together with serotonin formed locally by cells of several tissues, e.g., the lung, pancreas, and adipose tissue, a role in diverse physiological functions, such as regulation of tone of smooth muscle and promoting insulin production and lipogenesis, and exerts mitogenic effects on fibroblasts, adipocytes, smooth muscle cells, osteoblasts, and mesangial and endothelial cells [49,50,51,52,70,71,72]. It appears that, unlike the energy-wasting effects of serotonin of brain origin, the role of serotonin produced outside the brain promotes anabolic reactions, which may contribute to the development of obesity and metabolic syndrome (Section 9.5).

The various regulatory functions of serotonin in the immune system are important. Most immune cells, such as dendritic cells, monocytes, natural killer cells, B and T lymphocytes, mast cells, and eosinophils, synthesize serotonin or express 5-HT receptors. Especially investigated are the proinflammatory effects of serotonin, including recruitment of immune cells to the site of inflammation and increased production of ROS and inflammatory cytokines, which play a role in inflammatory bowel disease (IBD), irritable bowel syndrome, and celiac disease [70,73].

### 6.3. The Role of Melatonin

Melatonin plays a crucial role in regulating the circadian rhythm and exhibits antioxidant properties. Light inhibits melatonin synthesis in the pineal gland and retina; synthesis in other tissues is light-independent [74]. The effects of melatonin are mediated by two types of G-protein-coupled receptors (MT1 and MT2) located in various parts of the brain and peripheral tissues, such as the liver, pancreatic α and β cells, adrenal glands, blood vessels, and gonads [75].

#### 6.3.1. Melatonin and the Control of Circadian Rhythm

Melatonin is frequently referred to as the “hormone of darkness” because its production by pinealocytes increases at night, peaking between 2:00 and 4:00 a.m., and decreases during the day [74]. The nervous pathway involves the passage of signals from photoreceptors in the retina to the suprachiasmatic nucleus of the hypothalamus and then to the pineal gland. Once synthesized, melatonin is promptly released via the pineal recess into the cerebrospinal fluid of the third ventricle and into the bloodstream [74]. The diurnal fluctuation of melatonin production and its multifunctional biological effects enable the adjustment of diverse physiological functions, such as the sleep–wake cycle, hormone secretion (e.g., cortisol and leptin), anabolic and catabolic reactions, and reductions in body temperature and blood pressure, in response to the light–dark cycle. Currently, reduced melatonin production is a concern due to “darkness deficiency” from overexposure to artificial blue light [76].

#### 6.3.2. Melatonin as an Antioxidant

The indole moiety of the melatonin molecule is the reactive center of interaction with a variety of ROS and RNS to yield several metabolites, e.g., 6-hydroxymelatonin, 3-hydroxymelatonin, *N*^1^-acetyl-*N*^2^-formyl-5-methoxykynuramine, and *N*^1^-acetyl-5-methoxykynuramine. Unlike other antioxidants, such as vitamin C and E, several compounds derived from melatonin are also antioxidants. This phenomenon is referred to as the free radical scavenging cascade, which can scavenge up to four ROS [58,77]. Furthermore, melatonin has been shown to regulate the expression of transcription factors of various antioxidant enzymes [78].

#### 6.3.3. Other Melatonin Effects

Melatonin also exerts anti-inflammatory, immunomodulatory, neuroprotective, anti-aging, anti-carcinogenic, and anti-apoptotic functions [58,75]. Studies in rodents have demonstrated that melatonin signaling plays a crucial role in regulating retinal dopamine synthesis, rod/cone coupling, and protecting photoreceptors from oxidative stress and apoptosis [79].

## 7. TRP-KYN Pathway and Its Physiologic Importance

### 7.1. TRP-KYN Pathway

Approximately 85% of the TRP of food origin is degraded by the TRP-KYN pathway (Section 4). The key enzymes are TRP-2,3-dioxygenase (TDO) in the liver and indoleamine 2,3-dioxygenase (IDO), expressed primarily in immune cells. Under usual conditions, most of TRP is catabolized in the liver [80,81].

In addition to tissue expression sites, TDO and IDO differ in their regulation [80,81]. The TDO (K_m_ ~190 µM) expressed in the liver is activated by TRP supply and several hormones, primarily glucocorticoids [80,82,83]. Two isoforms of IDO (IDO1 and IDO2) have been identified in extrahepatic tissues. IDO1 with K_m_ ~20 µM is inducible by inflammatory stimuli, such as interferon γ (IFNγ), tumor necrosis factor α (TNFα), and interleukins 1 and 6 (IL-1 and IL-6). IDO2, with a high K_m_ value (~6800 µM), indicating its questionable role in humans, is constitutive [12,84].

**Figure 4:**
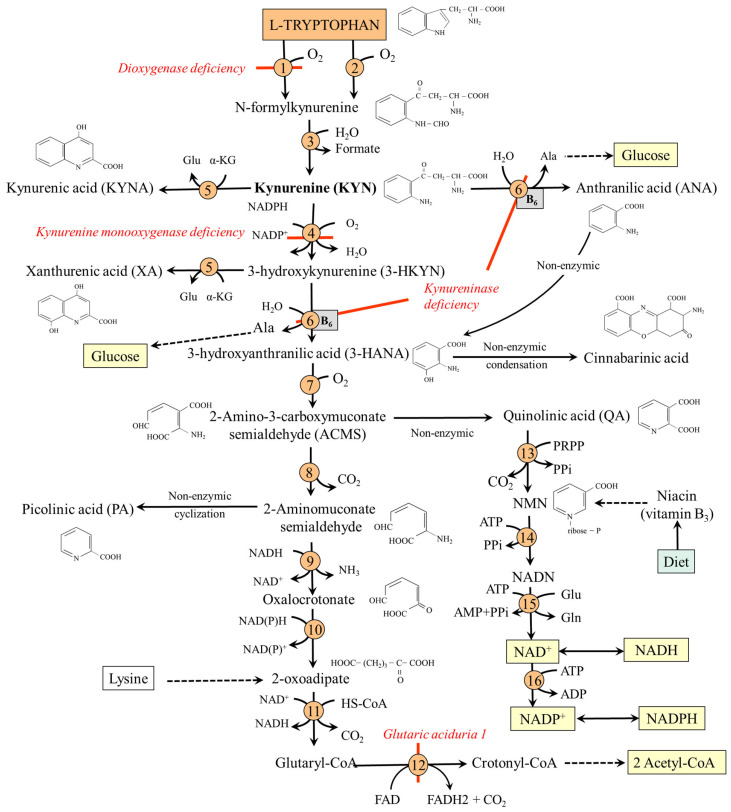
Kynurenine pathway of TRP metabolism. 1, Tryptophan 2,3-dioxygenase (pyrrolase); 2, indoleamine 2,3-dioxygenase; 3, kynurenine formylase (also called kynurenine formamidase); 4, kynurenine 3-monooxygenase; 5, kynurenine aminotransferase (several forms); 6, kynureninase; 7, 3-hydroxyanthranilate dioxygenase; 8, 2-amino-3-carboxymuconate semialdehyde decarboxylase (picolinate carboxylase); 9, 2-aminomuconate semialdehyde reductase; 10, oxalocrotonate reductase; 11, 2-oxoadipate dehydrogenase; 12, glutaryl-CoA dehydrogenase; 13, quinolinic acid phosphoribosyl transferase; 14, nicotinic acid mononucleotide adenylyl transferase; 15, NAD^+^ synthetase; 16, NAD^+^ kinase. Abbreviations: NADN, nicotinic acid adenine dinucleotide; NMM, nicotinic acid mononucleotide; PRPP, 5-phosphoribosyl-1-pyrophosphate.

*N*-formylkynurenine, formed by TDO or IDO, is converted by kynurenine formylase to KYN, which can be metabolized by several routes:

(i) Conversion to kynurenic acid (KYNA) by one of four aminotransferases. Of special importance is the high expression of type II in astrocytes of the brain, which directs the TRP-KYN pathway toward KYNA formation [85,86].
(ii) Synthesis of anthranilic acid (ANA) by kynureninase, which enables the bypass of the formation of 3-hydroxykynurenine (3-HKYN).
(iii) 2-amino-3-carboxymuconate-6-semialdehyde (ACMS) synthesis through 3-HKYN and 3-hydroxyanthranilic acid (3-HANA). The ACMS has two possible routes. First, non-enzymic conversion to quinolinic acid (QA), which is used by quinolinate phosphoribosyl transferase (QPRT) to form nicotinic acid mononucleotide (NMN), the precursor of NAD^+^ and NADP^+^. Second, decarboxylation to 2-aminomuconate-6-semialdehyde, which can be spontaneously converted to picolinic acid (PA), or oxidized via a sequence of reactions, shared with the lysine degradation pathway, to form two molecules of acetyl-CoA. Because TRP degradation through the TRP-KYN pathway yields acetyl-CoA and alanine, TRP is classified as both a glucogenic and a ketogenic amino acid (Figure 4).

#### Hereditary Disorders of the TRP-KYN Pathway

- *TDO deficiency*. The first human case without negative clinical consequences was described in 2017 [87]. Increased levels of TRP and serotonin characterize the biochemical phenotype.
- *Kynurenine 3-monooxygenase deficiency*. The disorder leads to the accumulation of KYN and a shift within the TRP-KYN pathway toward KYNA and ANA. The disease is associated with cognitive deficits [88].
- *Kynureninase deficiency (hydroxykynureninuria)*. It results in decreased synthesis of nicotinic acid mononucleotide and signs of pellagra. After TRP loading, patients excrete excessive amounts of XA, KYNA, 3-HKYN, and KYN [81].
- *Glutaric aciduria 1*. A rare autosomal recessive disease caused by glutaryl-CoA dehydrogenase deficiency. There is an increase in the levels of TRP and glutaryl-CoA derivatives, such as glutaric acid and glutarylcarnitine, and secondary carnitine deficiency. Increases also the concentration of lysine, which is also catabolized via glutaryl-CoA [89]. There is a risk of intellectual disability. Carnitine and choline supplementation, along with reduced lysine, TRP, and protein intake, is recommended [90].

### 7.2. Physiological Importance of the TRP-KYN Pathway

Under normal conditions, most of the TRP flux through the KYN-TRP pathway occurs in the liver via TDO, which is activated by TRP supply and cortisol. The TRP catabolism through IDO1 in extrahepatic tissues increases under various pathological conditions associated with immune system activation. Intermediates of the TRP-KYN pathway, collectively termed kynurenines, exert diverse biological functions and have been reviewed in detail by others [91,92]. In addition to kynurenines produced in the body, several intestinal bacteria produce them, which may act as AHR agonists and enter the bloodstream [93].

The extrahepatic tissues, including the brain and the microbiota, typically lack the complete set of enzymes involved in the TRP-KYN pathway [94]. Therefore, different metabolites can enter the pathway at various steps, and different products can be formed, which determines the role of the TRP-KYN pathway in a given tissue. In the liver, the TDO pathway plays a crucial role in controlling TRP levels in the body and as a source of NAD^+^ and NADP^+^. Pathways initiated by IDO1 in extrahepatic tissues have a unique role in various inflammatory conditions [2,3,95].

#### 7.2.1. The TRP-KYN Pathway and the Control of TRP Level in the Body

Under conditions of the surplus of exogenous TRP, the hepatic TDO is activated, and the flux through the TRP-KYN pathway increases. Oral administration of TRP to young women at daily doses from 1.0 to 5.0 g for 21 days increased urinary excretion of nicotinamide and KYN metabolites in proportion to TRP loading. Of eight TRP metabolites, 3-HKYN excretion had characteristics of a surrogate biomarker for excess TRP intake [96]. On the other hand, under conditions of TRP deficiency, the TDO is degraded by the ubiquitin-proteasome system, and TRP catabolism is inhibited [83].

#### 7.2.2. The TRP-KYN Pathway and Nicotinamide Nucleotide Synthesis

Nicotinamide nucleotides NAD^+^ and NADP^+^ are coenzymes for most dehydrogenases, which are key components of many oxidative and reductive pathways, including glycolysis, the citric acid cycle, and fatty acid oxidation. In humans, vitamin B_3_ (also known as niacin, the generic name for nicotinic acid and nicotinamide) is the primary substrate for NAD^+^ and NADP^+^ synthesis, and TRP is the alternative substrate [97].

The main site of nicotinamide nucleotide synthesis is the liver, from which the nucleotides are distributed to non-hepatic tissues. The flux towards nucleotides is ensured by high expression of TDO, 3-HANA dioxygenase, and QPRT, whereas ACMS decarboxylase is low (see Figure 4). It is generally accepted that 60 mg of dietary TRP is equivalent to 1 mg nicotinamide in humans [97]. High QPRT and low ACMS expression, resulting in effective nucleotide synthesis, have also been observed in some tumors [98]. The role of the kidneys is less significant due to high expression of ACMS decarboxylase, which directs TRP towards the synthesis of acetyl-CoA [99]. Small amounts of QA are metabolized to nicotinamide nucleotides due to the low specific activity of QPRT in the brain [100].

The importance of the TRP-KYN pathway in the synthesis of nicotinamide nucleotides is demonstrated by the consequences of impaired flux through the enzyme kynureninase, which requires vitamin B_6_ as a cofactor. Vitamin B_6_ deficiency or formation of its biologically inactive adduct with isoniazid, a drug for tuberculosis therapy, diverts TRP metabolism from production of nucleotides to the excessive formation of XA and KYNA. The symptoms are similar to pellagra [17].

#### 7.2.3. The TRP-KYN Pathway and the Immune System

Experimental studies suggest that increased flux through the TRP-KYN pathway, resulting from IDO1 activation, can, through AHR activation and TRP depletion in the local microenvironment, restrain tissue damage and exhibit anti-inflammatory, immunoregulatory, and pro-apoptotic effects. For instance, it has been shown that:

- Increased levels of KYN, PA, and QA inhibit the proliferation of T lymphocytes and natural killer (NK) cells [101].
- In piglets, 3-HKYN and 3-HANA have been shown to prevent allograft rejection and tubular injury in kidney transplantation [102].
- TRP depletion in a tissue due to increased flux through the TRP-KYN pathway induces, via a nutrient-sensing system termed the general control non-derepressable 2 (GCN2), proliferative arrest of cytotoxic T cells [103].
- KYNA exhibits antioxidant properties related to the ability to scavenge ROS [104]. On the contrary, QA stimulates iron-dependent lipid peroxidation and generation of ROS [105].
- KYN, 3-HKYN, and some of their derivatives protect the lens and the retina from UV irradiation. Their spontaneous deamination and binding to lens proteins contribute to age-related cataract [106].

#### 7.2.4. The TRP-KYN Pathway and the Nervous System

Besides TRP, some kynurenines, such as KYN, 3-HKYN, and ANA, primarily of hepatic and intestinal microbiota origin, cross the BBB and enter the TRP-KYN pathway. In contrast, QA, KYNA, and 3-HANA cross the BBB poorly [94]. Therefore, because brain activities of TDO and IDO are low, approximately 60% of flux through the TRP-KYN pathway is initiated by kynurenines, primarily KYN, which enter the brain from circulation [94].

Metabolites produced by the TRP-KYN pathway in the brain exert diverse, often contradictory effects. Attention is focused on a balance between KYNA and QA, given their opposing influences on NMDA receptors and the immune system. There is good evidence that KYNA, primarily formed by astrocytes due to high expression of kynurenine aminotransaminase II [86], acts as an NMDA receptor antagonist and exhibits both antioxidant and neuroprotective effects. In contrast, QA produced by microglia, i.e., immune cells of the central nervous system, is established as an NMDA receptor agonist with neurotoxic, pro-oxidative, and apoptotic effects resulting in neurodegeneration and lipid peroxidation [107,108,109,110].

## 8. TRP-IND Pathways

Approximately 10% of dietary TRP escapes absorption and utilization in the small intestine, becoming available to the microbiota of the large intestine (Section 4). Microbiota use TRP for the synthesis of proteins and various metabolites, which may exert both beneficial and detrimental effects on the host. The role of individual bacterial species capable of catabolizing TRP into indole and its derivatives has been examined in several review articles [5,6]. Relatively well known is the role of indoles due to their antioxidant properties and influence on intestinal motility, immunity, and gut barrier function, which is mediated through their ability to modulate the AHR and PXR. Less explored is the role of indoles in metabolic networks referred to as gut–tissue axes, e.g., gut–liver, gut–brain, and gut–renal, which contribute to systemic homeostasis and host health. Data on the levels of indole and its derivatives in feces, blood, and urine suggest that indole is the most abundant, followed by IAA and IPA [6].

**Figure 5:**
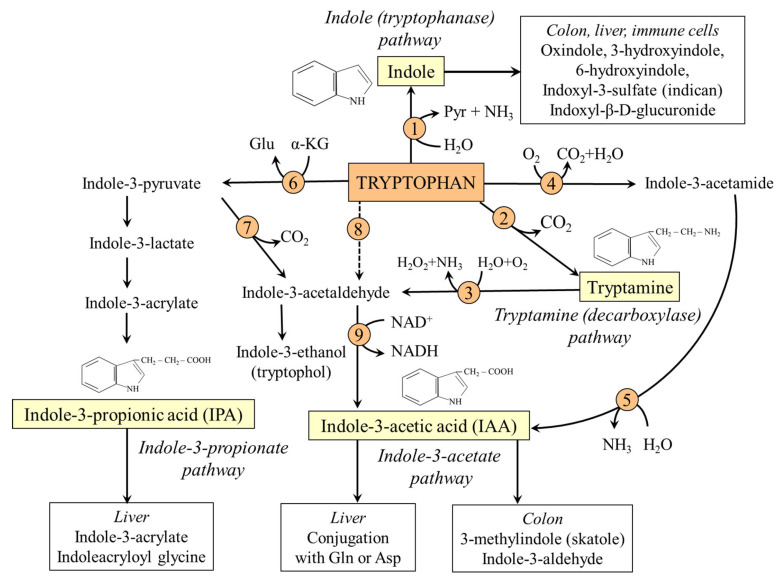
TRP-IND pathways in microbiota in the large intestine. 1, Tryptophanase; 2, tryptophan decarboxylase; 3, monoamine oxidase; 4, tryptophan 2-monooxygenase; 5, indole-3-acetamide hydrolase; 6, aromatic amino acid transaminase; 7, indole-3-pyruvate decarboxylase; 8, oxidoreductase, dehydratase, and nitrilase; 9, indole-3-acetaldehyde dehydrogenase. Gln, glutamine; Asp, aspartic acid.

This section examines four pathways of TRP catabolism that lead to the formation of substances with an unbroken indole ring, thereby referred to as TRP-IND pathways (Figure 5).

### 8.1. Tryptophanase (Indole) Pathway

Many bacteria, including the genera *Escherichia*, *Clostridium*, and *Bacteroides*, hydrolyze TRP by tryptophanase (L-tryptophan indole-lyase) to indole, which exerts beneficial effects on the host through its antioxidant properties and influence on AHR and PXR [5,6]. Moreover, in enteroendocrine L cells of the gut, indole stimulates the secretion of incretin glucagon-like peptide-1 (GLP-1), which increases insulin secretion, suppresses appetite, and slows gastric emptying [111]. In the colon, indole can be converted into several compounds, including oxindole, 3-hydroxyindole (also known as indican), and 6-hydroxyindole. These substances, together with other indole derivatives such as indole propionate, indole acetate, and indole lactate, are responsible for stool odor.

The liver plays a primary role in degrading absorbed indole and its derivatives into soluble compounds, which can be excreted in the urine. Cytochrome P450 enzymes oxidize indole to oxindole, 6-hydroxyindole, or 3-hydroxyindole (indoxyl), which is conjugated by sulfotransferases to indoxyl-3-sulfate. The increased excretion of this substance in the urine (determined as indican) is a marker of leaky gut syndrome, a disorder of the intestine’s barrier function [112,113].

### 8.2. Decarboxylation (Tryptamine) Pathway

Tryptamine (indolethylamine), produced by TRP decarboxylation in the genera *Clostridium* and *Ruminococci,* stimulates through 5-HT_4_ receptors serotonin secretion by ECC, activates AHR of immunocytes and intestinal cells, and inhibits the formation of proinflammatory cytokines, such as TNF-α [5,6]. Tryptamine readily enters the circulation and is able to cross the BBB, where it activates 5-HT and trace amine-associated receptors, and acts as a potent agent releasing monoamines, i.e., serotonin, dopamine, and norepinephrine [114]. After conversion to an aldehyde by MAO in colonic epithelium, brain, or liver, it enters the indole-3-acetate pathway (Figure 5).

### 8.3. Indole-3-Propionate Pathway

Indole-3-propionic acid (IPA) is produced primarily by *Clostridium*, *Peptostreptococcus*, and *Ruminococcus* families [5,6]. IPA is a potent free radical scavenger, agonist of AHR and PXR, an anti-inflammatory substance, and enhances gut barrier integrity through expression of tight junction proteins [115,116]. Significant amounts of IPA of gut origin have been found in CSF, and it is supposed that IPA protects microglia from inflammatory stimuli and boosts the level of KYNA [116,117]. A diet high in lipids and low in fiber reduces IPA production, whereas a diet high in vegetables increases it [118].

### 8.4. Indole-3-Acetate Pathway

A common intermediate in various pathways of TRP degradation is indole-3-acetaldehyde, which is oxidized to indole-3-acetic acid (IAA) by bacteria that express indole-3-acetaldehyde dehydrogenase, such as the genera *Lactobacillus*, *Clostridium*, *Bifidobacterium*, and *Bacteroides* [6]. Unclear is the possibility of IAA synthesis via TRP monooxygenase and indole-3-acetamide hydrolase, enzymes playing a crucial role in the biosynthesis of plant hormones [119]. The terminal products of the IAA pathway in the gut are skatole and indole-3-aldehyde. In the liver, IAA is conjugated with glutamine or aspartate or oxidized by cytochrome P450 to several metabolites [120].

Besides the colon, IAA can be found in plasma and the brain, and other tissues. Compared with IPA, the mechanism of action and role in homeostasis are less clear. IAA acts as a ligand to AHR, induces ROS generation, transcription of proinflammatory cytokines, apoptosis, loss of membrane integrity, and necrosis [113]. It has been shown that there is a direct correlation between IAA’s cytotoxic effect and cells’ peroxidase activity. Neutrophils, which exhibit higher peroxidase activity, are more sensitive to the cytotoxic effects of IAA than lymphocytes or macrophages, in which enzyme activity is low [121]. IAA concentration in plasma increases in patients with uremia, and it is classified as a uremic toxin (Section 9.7).

## 9. Alterations in Tryptophan Metabolism Under Different Physiological and Pathological Conditions

This section is devoted to disorders of TRP metabolism that can develop due to alterations in TRP intake, aging, alcoholism, and several diseases. Congenital disorders of TRP metabolism have been mentioned in previous parts of this article.

### 9.1. Dietary TRP Deficiency

TRP is a nutritionally essential and proteinogenic amino acid, and its long-term dietary deficiency results in a negative protein balance [122]. However, TRP differs from other essential amino acids in its role in numerous biologically important functions. Therefore, the symptoms of TRP deficiency are primarily related to disturbances in its degradation pathways.

- *TRP-SER pathway*. In rats, administration of a TRP-free amino acid mixture resulted in a sharp drop in blood TRP and decreased levels of TRP, serotonin, and 5-HIAA in the brain [123]. In humans, the TRP-free amino acid mixture caused, within 4 h after ingestion, a substantial decrease in plasma TRP associated with depression and anxiety [64]. In a study examining the differences in anxiety, depression, and mood in healthy adults after consuming a high and a low TRP diet for four days each, a diet with high content of TRP resulted in fewer depressive symptoms and decreased anxiety [124]. In summary, the lack of TRP in the body can result in depression and anxiety due to insufficient serotonin production in the brain [125,126].
- *TRP-KYN pathway*. The TRP-KYN pathway is an important source of nicotinamide nucleotides. Prolonged deficiency of TRP and niacin (vitamin B_3_, i.e., nicotinic acid and nicotinamide), also referred to as vitamin PP (pellagra preventive), results in pellagra, the photosensitive disease that has been common in populations where corn was the staple food. Maize contains low amounts of TRP, and the majority of niacin is bound to polysaccharides as niacytin, which cannot be hydrolyzed by the mammalian digestive system [17]. The main symptoms of pellagra are described as the “3 Ds”: dementia, diarrhea, and dermatitis. New corn varieties have higher levels of both niacin and TRP. The symptoms of pellagra have also been observed in cases of non-nutritional origin of TRP deficiency, e.g., Hartnup’s disease (Section “Hereditary Disorders of TRP Transport Through the Plasma Membrane”) and carcinoid, serotonin-producing tumor originating from ECC [17].
- *TRP-IND pathway*. Experimental studies have clearly demonstrated that TRP dietary deficiency leads to dysbiosis, which in turn promotes the development of health problems in the host. In rats, a TRP-free diet decreased IPA concentration in stool and blood [127]. In a mouse model, TRP deficiency induced gut microbiota dysbiosis, altered the formation of various gut metabolites and expression of regulatory T-lymphocytes, and increased proinflammatory cytokine levels [128,129].

### 9.2. TRP and Aging

The course of aging is influenced by genetic, lifestyle, and environmental factors, resulting in morphological and functional changes in the body that impact the quality of life and can increase the risk of disease and mortality. Sometimes, it is not easy to determine which alterations are due to old age and which are due to poor lifestyle or disease (pathological aging).

- *TRP-SER pathway*. Significant alterations occur in melatonin synthesis. Melatonin levels decline gradually over the lifespan and may be related to decreased sleep efficacy, as well as to the deterioration of many circadian rhythms and antioxidant defense [76]. Therefore, melatonin supplementation should be considered in the elderly.
- *TRP-KYN pathway*. Aging is associated with increased activity of the TRP-KYN pathway due to upregulated cortisol production, an activator of TDO, and the presence of proinflammatory cytokines, which induce IDO [80]. A trend toward reduced TRP and increased kynurenine levels, primarily KYN, KYNA, and QA, has been observed in serum and CSF in older individuals [130]. Kynurenines are supposed to play a role in alterations in cognitive function and depression in aging [130]. For these reasons, it is unclear whether TRP supplementation should be recommended in old age, even though TRP levels tend to decline. In addition, a causal link between downregulation of KYN formation and lifespan prolongation in vertebrates has been suggested [131].
- *TRP-IND pathways*. Aging and age-related disorders are influenced by substances of gut microbiota origin that appear in the blood, such as endotoxins, ammonia, and indoles. Some indole derivatives, particularly IPA, cross the BBB and exert neuroprotective effects [132,133]. In muscles, indoles can slow the progression of sarcopenia, i.e., the loss of skeletal muscle associated with aging, by inhibiting the production of proinflammatory cytokines, such as TNF-α, which activate proteolysis and amino acid oxidation [134,135]. Therefore, the gut microbiome is a target of studies examining the possibility of optimizing its composition to form beneficial metabolites and slow down the development of undesirable consequences of aging [136,137,138].

### 9.3. TRP and Alcoholism

Chronic drinking of alcohol has many negative social consequences and can damage several organs, especially the liver and the brain. Changes in TRP metabolism probably contribute to their pathogenesis.

- *TRP-SER and TRP-KYN pathways*. Acute alcohol intake activates TDO and TRP degradation via the TRP-KYN pathway in the liver, reducing circulating TRP availability to the brain and decreasing serotonin and melatonin synthesis [139]. Serotonin deficit may contribute to alcohol-induced aggression, depression, and impaired memory. The suppression of melatonin synthesis contributes to the development of sleep disorders [140].
  Alterations in TRP metabolism probably also play a role in a variety of neuropsychiatric symptoms in individuals who try to abstain from alcohol. In rats fed an ethanol-containing diet, alcohol withdrawal increased corticosterone concentrations associated with TDO activation, resulting in decreased concentrations of TRP and serotonin synthesis in the brain [141]. A recent study performed at the 5th and 10th day after alcohol withdrawal in patients with alcohol-use disorder demonstrated increased KYN/TRP ratio and QA concentration, which exerts neurotoxic effects, but not KYNA, which possesses neuroprotective properties [142]. Therefore, it may be hypothesized that the disruption of TRP metabolism contributes to alcohol-related neuropathy and myopathy, which is frequent in subjects who consume alcohol chronically [143].
- *TRP-IND pathways*. Alcohol consumption alters microbiota composition, TRP metabolism through the TRP-IND pathway, and host immunity. Dysbiosis and decreased intestinal levels of IAA have been observed in chronic-binge ethanol-fed mice, which were associated with reduced production of interleukin-22 by innate lymphoid cells in intestinal lamina propria [4].

### 9.4. TRP and Inflammatory Bowel Disease

Inflammatory bowel disease (IBD) is a group of diseases of the gastrointestinal tract characterized by repetitive episodes of inflammation associated with abdominal pain, diarrhea, rectal bleeding, tiredness, and weight loss. The main forms include ulcerative colitis and Crohn’s disease. An important role in the pathogenesis of IBD plays dysbiosis, resulting in altered production of various compounds, which can exert both detrimental and beneficial influences on disease progression. The expression of AHR, which helps maintain mucosal homeostasis, is defective in both forms of IBD [10]. Both disorders have a genetic predisposition and increase the risk of colorectal cancer.

- *TRP-SER pathway*. Upregulation of ECC number and TRPH1 expression, as well as increased gut and plasma serotonin levels, have been demonstrated in patients with IBD [144,145,146]. Decreased expression of the serotonin transporter is likely also a contributing factor to increased mucosal serotonin signaling [146,147]. Pharmacological blocking of 5-HT receptors and peripheral serotonin synthesis using a TRPH inhibitor has been shown to attenuate intestinal inflammation in experimental models [145,148,149].
- *TRP-KYN pathway*. Increased expression of IDO1 in colonic biopsies and elevated levels of kynurenines, primarily QA, have been demonstrated in patients with IBD. Since QA exhibits proinflammatory properties, its increase may contribute to disease exacerbation [150].
- *TRP-IND pathways*. Unlike serotonin and KYN metabolites, some researchers suggest that indole metabolites may hold therapeutic potential [151]. IPA has been shown to suppress experimental colitis in mice [152]. Indole-3-carbinol, an indole derivative found in vegetables, has been found to prevent colitis in mice [153]. Studies in murine and porcine models of colitis demonstrated that TRP supplementation enables, via AHR, the homing of regulatory T cells to the large intestine and reduces the risk of colitis [154,155]. Taken together, the findings suggest that TRP administration, accompanied by a simultaneous adjustment of the microbiome to favor indole production, can have a therapeutic effect.

### 9.5. TRP and Metabolic Syndrome

Metabolic syndrome refers to a group of individuals who are at a higher risk of death due to developing serious illnesses, primarily cardiovascular diseases, type 2 diabetes mellitus (T2DM), and cancer. General characteristics include insulin resistance, abdominal obesity, hypertriglyceridemia and hypercholesterolemia, hypertension, and dysbiosis. Systemic low-grade inflammation, primarily originating from adipose tissue infiltrated by immune cells, and endotoxemia, resulting from impaired gut barrier integrity, play a crucial role in the pathogenesis [156]. In most conditions, all TRP metabolic pathways are dysregulated.

- *TRP-SER pathway in the periphery*. Gut-derived serotonin is an important driver of the development of metabolic syndrome. Serotonin can promote obesity and nonalcoholic fatty liver disease (NAFLD) by stimulating insulin secretion, inhibiting thermogenesis in beige adipose tissue, and increasing lipogenesis in white adipose tissue and the liver [71,72]. Increased serotonin formation, resulting from higher ECC density and TRPH1 expression in the small intestine, has been demonstrated in rodent models of obesity [157,158]. In humans, elevated serotonin concentrations have been reported in hypertension, atherosclerosis, and arterial thrombosis [159]. TRPH inhibitors that decrease peripheral serotonin synthesis are being investigated in the treatment of diseases associated with metabolic syndrome [50,160].
- *TRP-SER pathway in the brain*. In the brain, the flux through the TRP-SER pathway decreases somewhat due to decreased TRP availability [161,162,163]. The cause is not a decrease in plasma TRP level but rather an increase in BCAAs, which compete with TRP for the L-transporter. The BCAA level increases due to insulin resistance [30]. The consequences of decreased flux through the TRP-SER pathway in the brain may include sleep and diurnal rhythm disorders, depression, increased food intake, and decreased energy expenditure. A systematic review and meta-analysis have demonstrated that short sleep duration is associated with an increased risk of metabolic syndrome [161]. Other studies have demonstrated that increased dietary TRP intake had beneficial effects on sleep duration and plasma biomarkers of metabolic syndrome [162].
- *TRP-KYN pathway*. The flux through the TRP-KYN pathway increases due to IDO1 induction by chronic inflammation. Increased levels of KYN metabolites or KYN/TRP ratio have been observed in most disorders associated with metabolic syndrome, including obesity [163,164,165], T2DM [166], and cardiovascular events [167].
- *TRP-IND pathways*. Studies in subjects with metabolic syndrome have demonstrated decreased levels of indole and its derivatives in plasma and feces, a shift from the TRP-IND to the TRP-KYN pathway in the gut, and intestinal inflammation and disruption of the intestinal barrier [136,165]. It has been suggested that decreased levels of IPA, which exerts benefits on gut homeostasis through AHR and PXR, can predict the risk of NAFLD, T2DM, and cardiovascular disease [168].

### 9.6. TRP and Diseases of the Nervous System

All three pathways of TRP metabolism can play a role in the pathogenesis of psychiatric (e.g., schizophrenia and depression) and neurodegenerative diseases (e.g., dementia, Huntington’s disease, multiple sclerosis, and Alzheimer’s and Parkinson’s diseases). There are dozens of experimental and clinical studies investigating TRP metabolism in nervous system diseases, which reveal distinct patterns of TRP metabolism dysregulation across various brain regions. Unfortunately, their analysis goes beyond the scope of this article. I will highlight common pathogenic features, particularly the role of neuroinflammation and alterations in the TRP-KYN pathway. Recent articles dedicated to TRP metabolism in specific disorders are cited.

- *TRP-SER pathway*. Serotonin depletion is the leading cause of a mental disorder, referred to as major depressive disorder, characterized by chronically pervasive low mood, low self-esteem, and loss of interest in usual activities [169,170]. The cause of decreased flux through the TRP-SER pathway is likely IDO1 activation in microglia, driven by neuroinflammation, leading to decreased TRP availability for serotonin synthesis. The consequence is also a decreased formation of *N*-acetylserotonin and melatonin, resulting in disturbances in sleep, increased vulnerability of the central nervous system to oxidative stress, and the development of neurodegenerative diseases [169].
- *TRP-KYN pathway*. Neuroinflammation and subsequent IDO1 activation by various inflammatory mediators play a pivotal role in dysregulating the TRP-KYN pathway in most diseases of the nervous system. Decreased levels of KYNA and increased QA, or a decreased KYNA-to-QA ratio, in CSF, brain, or plasma have been reported in Alzheimer’s disease [130], Parkinson’s disease [130,171], Huntington’s disease [172], and multiple sclerosis [173,174,175]. Post-mortem studies revealed significantly increased activity of 3-HANA dioxygenase and elevated levels of QA in the cortex and striatum of patients with Huntington’s disease [176]. KYNA negatively correlated with depression severity and significantly increased after therapy [170].
  Unlike the decreased KYNA to QA ratio in depression and neurodegenerative diseases, elevated levels of KYNA probably play a role in the pathogenesis of schizophrenia. Increased KYNA levels and downregulated kynurenine 3-monooxygenase gene expression have been found in the brains of people with schizophrenia [177,178,179]. The hypothesis aligns with a theory that the hypofunction of NMDA receptors is a component of the disease’s pathophysiology [180].
- *TRP-IND pathways*. Several investigators have demonstrated that indoles produced by gut microbiota from TRP play a role in the development and function of the nervous system, as well as in the pathogenesis of its diseases [117,132,133]. It is assumed that most naturally occurring indoles in the blood enter the brain and exert neuroprotective effects, primarily by mitigating oxidative stress [133]. Special attention is focused on IPA, which acts as a free radical scavenger and an anti-inflammatory substance, thereby decreasing the production of proinflammatory cytokines [117].

### 9.7. TRP and Chronic Renal Insufficiency

Chronic renal insufficiency (CRI) results from serious kidney diseases such as glomerulonephritis, pyelonephritis, and diabetic nephropathy. The primary alterations include increased concentrations of urea, creatinine, and potassium, as well as metabolic acidosis. The accumulation of waste products in the body is responsible for various clinical manifestations, including nausea, vomiting, loss of appetite, fatigue, dyspnea, muscle cramps, cognitive dysfunction, encephalopathy, seizures, and coma in the final stage. A special role in the pathogenesis of CRI is played by dysbiosis and compromised intestinal barrier integrity, resulting in increased exposure of the body to pathogenic bacteria and endotoxins.

The focus of nephrologists’ attention is on substances collectively called uremic toxins, including metabolites of TRP-KYN and TRP-IND pathways, which accumulate in patients with renal failure. These substances, through different mechanisms, such as activation of AHR, NF-κB, and MAPK, induce oxidative stress, accelerate renal disease progression, contribute to endothelial dysfunction, and increase the risk of cardiovascular diseases and thrombosis [181,182,183,184]. Because several uremic toxins, such as indoxyl sulfate and IAA, are bound to plasma proteins, they are difficult to remove by haemodialysis. Therefore, compounds that can displace protein-bound toxins, such as ibuprofen and warfarin, are investigated to improve the clearance of uremic toxins by dialysis [185].

- *TRP levels and the TRP-SER pathway*. Decreased total and protein-bound TRP levels are found in subjects with CRI. In contrast, concentrations of free TRP are usually increased or unaltered due to TRP replacement at the binding site of albumins by uremic toxins [112,186]. An important alteration in aminoacidemia is a decrease in the concentration of most essential amino acids, primarily BCAA (valine, leucine, and isoleucine), due to acidosis-induced oxidation in muscles [187]. An increased free TRP to BCAA ratio can enhance TRP entry into the brain and serotonin production, and play a role in uremic anorexia [188].
- *TRP-KYN pathway*. The activation of TDO and IDO1 by cortisol and proinflammatory cytokines, as well as impaired renal function, are the leading causes of elevated kynurenine levels in patients with uremia [189]. A role also plays the suppression of QA utilization in NAD^+^ synthesis, as demonstrated in kidney biopsies from patients with CRI [190]. The kynurenines recognized as uremic toxins include KYN, KYNA, ANA, 3-HKYN, 3-HANA, and QA. However, their role in uremia is poorly understood. Relatively well-documented is the brain toxicity of QA [182,191,192]. Experiments conducted in vitro also indicate that QA inhibits erythropoietin gene expression, contributing to the pathogenesis of uremic anemia [193]. Experimental studies indicate increased entry of some kynurenines into the brain due to the BBB disruption. In rats with CRI, plasma and brain TRP levels were decreased, while KYN and 3-HKYN levels were elevated [194].
- *TRP-IND pathways*. Concentrations of both free and protein-bound indole metabolites, primarily IAA, indoxyl sulfate, and indoxyl-β-D-glucuronide, recognized as uremic toxins, increase in patients with CRI due to impaired gut barrier integrity and their decreased elimination in urine [112,113]. Unlike the positive influence of most indole derivatives in the gut, IAA, indoxyl sulfate, and indoxyl-β-D-glucuronide act in cells of the cardiovascular system as pathogenic agents that, via the AHR, induce the transcription of proinflammatory cytokines, apoptosis, and oxidative stress. Their increased concentrations correlate with cardiovascular events, such as atherosclerosis and thrombosis [113,195]. The therapeutic potential of orally administered spherical carbon adsorbent AST-120 is investigated, which reduces the absorption of indoles from the gut and indoxyl-sulfate levels in plasma [196].

### 9.8. TRP and Liver Cirrhosis

Liver cirrhosis, the end-stage of various forms of chronic liver injury, such as hepatitis, NAFLD, and biliary cholangitis, is characterized by hepatocyte necrosis, regenerative nodules, and fibrosis. Cirrhosis occurs in two stages—compensated and decompensated. In the compensated stage, the symptoms of liver damage are not pronounced. A broad spectrum of symptoms, including jaundice, blood coagulation disorders, muscle wasting, amino acid imbalance, hyperammonemia, and encephalopathy, is present in the decompensated stage. A significant role in liver cirrhosis development is played by inflammatory signals that activate ROS and RNS formation in Kupffer cells, as well as the stimulation of hepatic stellate cells, also referred to as Ito cells or fat-storing cells, which are involved in collagen secretion and the development of liver fibrosis. In liver cirrhosis, all pathways of TRP metabolism are altered.

- *TRP levels and the TRP-SER pathway*. An increased concentration of free TRP is a well-documented finding in patients with liver cirrhosis [26,197]. Primary causes are impaired TRP catabolism via the TRP-KYN pathway in the liver, due to reduced hepatocyte mass and portacaval shunts. A role has also decreased the amount of albumin-bound TRP as a result of hypoalbuminemia and increased concentration of free fatty acids and indoles, which compete with TRP for the binding site. On the other hand, the BCAA level (valine, leucine, and isoleucine) in cirrhosis decreases due to their extensive use for ammonia detoxification to glutamine in muscles [198,199]. Because TRP and BCAA share the same carrier, an increased TRP-to-BCAA ratio enhances TRP availability for serotonin synthesis in the brain. It may contribute to the pathogenesis of anorexia and poor nutritional status in some patients [29,35,36]. There is probably no direct relationship between TRP levels and encephalopathy. Oral TRP load increased plasma TRP levels but did not induce or worsen signs of hepatic encephalopathy [200].
  Data on changes in plasma serotonin and its role in the periphery are inconsistent. Several studies have reported a decrease in platelet-bound serotonin associated with thrombocytopenia and blood coagulation disorders [201,202].
- *TRP-KYN pathway*. Increased concentrations of kynurenines, primarily due to extrahepatic IDO1 induction, have been found in plasma and CSF in patients with liver disease [203,204]. Their role in cirrhosis is controversial. There are reports that the immunosuppressive effects of some kynurenines protect against viral hepatitis and reduce oxidative stress and inflammation. On the other hand, immunosuppression can contribute to multiorgan damage and promote the development of nosocomial infections and carcinogenesis [204,205,206]. A growing body of evidence suggests that neuroinflammation and TRP-KYN pathway dysregulation contribute to the pathogenesis of encephalopathy. Increased production of neurotoxic metabolites, 3-HKYN and QA, has been observed in animal models and in humans with hepatic encephalopathy [203,207,208].
- *TRP-IND pathways*. Disrupted intestinal barrier integrity and dysbiosis, usually overgrowth of pathogenic genera *Staphylococcus*, *Enterococcus*, and *Enterobacter* are common findings in subjects with liver cirrhosis [137,209]. The result is increased entry of indoles and other microbial products, such as ammonia and endotoxin, into portal circulation. The inability of the cirrhotic liver to clear such compounds results in their increased levels in systemic circulation and influence on the host. It is a consensus that dysbiosis and “leaky gut syndrome” are risk factors for decompensation of the hepatic disease. Unfortunately, data on the amounts and spectrum of indoles formed in the gut in cirrhosis are absent, and their effects on the pathogenesis of liver cirrhosis are not entirely clear. It has been shown that oxindole, formed in the liver from indole by cytochrome P450, crosses the BBB and is apparently involved in the pathogenesis of hepatic encephalopathy [210,211].

### 9.9. TRP and Cancer

Alterations in TRP metabolism play a role in both tumor growth and the host response. Activation of the TRP-KYN pathway appears to play a significant role in most cancer types. TRP-SER and TRP-IND pathways play unique roles in colorectal cancer.

- *TRP-SER pathway in the brain*. Studies in subjects with cancer demonstrated both decreased and increased plasma concentrations of free TRP, suggesting alterations in its entry into the brain and serotonin synthesis, which can play a role in behavior, mental functions, and onset of anorexia-cachexia syndrome [212,213,214,215].
- *TRP-SER pathway in the periphery*. Serotonin has been shown to activate cancer cell proliferation, differentiation, and migration, and angiogenesis in various types of cancer [215,216,217]. The carcinogenic effect is mediated primarily through autocrine serotonin signaling affecting various types of 5-HT receptors depending on the type and stage of cancer [217]. For example, increased expression of TRPH1 and 5-HT_7_ receptors has been reported in breast cancer [216]. Furthermore, serotonin activates RhoA/ROCK/YAP signaling and promotes colon carcinogenesis via serotonylation [218]. In contrast to the carcinogenic potential of serotonin, 5-methoxytryptophan, a byproduct of the TRP-SER pathway, referred to as cytoguardin (see Figure 3 and Section 11.1), likely acts against cancer growth [219,220].
  In connection with the role of the TRP-SER pathway in cancer, carcinoid, a tumor originating from the ECC, that produces 5-hydroxytryptophan and serotonin, should be mentioned. Clinical manifestations include decreased TRP levels, signs of pellagra due to reduced synthesis of nicotinamide nucleotides via the TRP-KYN pathway, paroxysmal facial flushing, diarrhea, bronchospasm, and heart valve disease. A part of the therapy is the TRPH1 inhibitor, teloristat ethyl [62].
- *TRP-KYN pathway*. Increased expression of IDO1 and TDO, as well as increased activity of the other enzymes in the TRP-KYN pathway, have been reported in various types of cancer, including breast, stomach, colon, pancreatic, and lung cancers [214,221,222]. Notably, QPRT, the enzyme directing the TRP-KYN pathway towards NAD^+^ generation, was upregulated in invasive breast cancer and aggressive glioblastomas [98].
  It is a consensus that increased kynurenine formation contributes to immune suppression in the tumor microenvironment, as well as neovascularization, tumor growth, and metastasis [11]. The progression of cancer also promotes systemic immune suppression, primarily resulting from the upregulation of IDO1 by host dendritic cells in tumor-draining lymph nodes [223]. The mechanism by which cancer-induced TRP catabolism leads to immunosuppression in the host is unclear; the role of Treg lymphocyte-associated antigen 4 (CTLA-4) has been hypothesized [224]. Preclinical models have demonstrated that inhibiting IDO1, TDO, and kynurenine 3-monooxygenase can enhance the efficacy of cytotoxic chemotherapy and radiotherapy in various types of cancer [214,222,225].
- *TRP-IND pathways*. Our understanding of the role of indole derivatives in cancer remains limited. Several experimental studies indicate their cytostatic and preventive effects [226,227,228]. For example, an AHR agonist, indole-3-carbinol, decreased viability and accelerated apoptosis in cultures of human colorectal carcinoma cell lines [228].

## 10. Tryptophan as a Dietary Supplement

TRP-enriched food and TRP-containing supplements can prevent pellagra in populations consuming TRP and vitamin B_3_-deficient diets (Section 9.1). On the other hand, in developed countries, TRP has been widely used as an over-the-counter substance for the management of depression, headaches, insomnia, and hyperactivity. The most frequently recommended daily dose is 3 g [6,124]. TRP supplementation is also supposed to increase the formation of several beneficial indole metabolites. It has been suggested as a non-invasive therapy to prevent the onset or relapse of IBD and age-related disorders [154,155,229].

However, in some studies focused on mood and behavior, administration of a relatively high dose of TRP had no effect [96]. Controversial are also the results of clinical trials examining the supposed beneficial effects of TRP on social behavior in people prone to aggression and schizophrenia [230,231,232,233]. The key to explaining inconsistent observations may lie in the influence of several factors on TRP availability for serotonin synthesis in the brain. The primary ones are likely the catabolism of administered TRP through the TRP-SER pathway in the gut and the TRP-KYN pathway in the liver, as well as the plasma ratio of free TRP to LNAA (see Section 3.3 and Section 3.4).

### Risks and Side Effects of TRP Administration

TRP supplementation is generally considered safe. Although TRP has been studied for six decades, few side effects, including tremor, nausea, and dizziness, have been reported [231]. Administration of up to 5 g of TRP per day for three weeks had no adverse effect in healthy women; based on the 3-HKYN biomarker, a tolerable upper intake level (the highest average daily nutrient intake level that is likely to pose no risk of adverse health effects to most individuals in the general population) was suggested at 4.5 g/d for TRP [96].

Caution should be exercised when supplementing TRP with drugs that affect serotonin metabolism. Life-threatening “serotonin syndrome”, which includes neuromuscular abnormalities and autonomic hyperactivity, can occur under the condition of simultaneous administration of TRP and serotonin reuptake or MAO inhibitors [234]. In this context, it should be noted that the so-called eosinophilia-myalgia syndrome, which emerged at the end of the 1980s following the intake of dietary supplements containing TRP, was caused by bacterial contamination of the product [235].

There are various side effects, which have been evaluated only sporadically. First of all, TRP administration to exploit the benefits of its specific metabolites may lead to unwanted effects due to increased flux through other metabolic pathways. For instance, TRP administration for boosting serotonin levels in the brain, e.g., for depression therapy, may cause serious adverse side effects through increased serotonin synthesis in the periphery. In particular, the concern should be serotonin’s mitogenic effects [49,50,51], which could promote tumor growth. It has been demonstrated that long-term serotonin administration induces heart valve disease in rats [236]. Adverse side effects may also occur due to competition for the amino acid transporter at the plasma membrane. Specifically, high doses of TRP can affect the transport of LNAA through the B^0^ and L systems, their appearance in the blood, transport across the BBB, and supply for protein synthesis [237].

TRP supplementation can be detrimental in subjects with liver cirrhosis and renal injury. In liver cirrhosis, TRP supplementation could result in a marked increase in the TRP to BCAA ratio, resulting in anorexia and poor nutritional status due to increased serotonin formation and impaired BCAA transport into the brain and muscles [29,35,36]. It is a consensus that several TRP metabolites, such as KYN, 5-HIAA, and indoxyl sulfate, exacerbate kidney damage by activating the AHR signaling pathway (Section 9.7). It can therefore be assumed that TRP supplements will increase flux through the TRP-KYN and TRP-IND pathways, increase the production of uremic toxins, and worsen the disease.

## 11. Therapeutic Possibilities of Targeting Individual Pathways of Tryptophan Metabolism

The administration of specific metabolites and the modulation of enzymes, transporters, and receptors involved in individual TRP metabolism pathways is being investigated as a new therapeutic approach for the treatment of various diseases. Targeting the specific TRP metabolic pathway can be more effective than TRP therapy, as it avoids the adverse effects of increased TRP flux through unwanted pathways. Note that the list of possibilities is not exhaustive, and more detailed information can be obtained from several excellent reviews acknowledged in this section.

### 11.1. Targeting the TRP-SER Pathway

Efforts to influence the TRP-SER pathway are targeted both at its role in the brain and the periphery. The goal of influencing the TRP-SER pathway in the brain is primarily to treat depression and insomnia; the goal in the periphery is particularly the treatment of IBD and cancer.

- *5-Hydroxytryptophan*. 5-hydroxytryptophan, the intermediate in the TRP-SER pathway, crosses the BBB, and, unlike TRP, it cannot be shunted into niacin or protein synthesis. Its administration can affect serotonin levels in both the brain and the periphery. It has shown good therapeutic potential for depression therapy when used with selective serotonin reuptake inhibitors [238,239]. Positive effects have also been reported in the treatment of headaches, fibromyalgia, anxiety, insomnia, and as an anorectic [238,239].
- *Melatonin*. Melatonin is both water- and lipid-soluble (‘amphiphilic’) and can freely cross plasma membranes, including the BBB. Therefore, melatonin and several melatonin analogues (e.g., ramelteon, agomelatine, and tasimelteon) are currently used to treat sleep disorders, prevent desynchronosis (jet lag), as an antioxidant, and in other conditions [76]. Current evidence shows that melatonin protects against liver injury and inhibits the progression of liver cirrhosis [240]. The recommended dose has not been clearly established and varies from units to hundreds of mg daily [76,241].
- *N-acetylserotonin (normelatonin)*. *N*-acetylserotonin, the intermediate in endogenous synthesis of melatonin from serotonin, and its derivative *N*-(2-(5-hydroxy-1*H*-indol-3-yl) ethyl)-2-oxopiperidine-3-carboxamide (HIOC) act as agonists of melatonin receptors and potent antioxidants. Both are investigated as potential therapeutic agents for brain injury, autoimmune encephalomyelitis, ischemic encephalopathy, and other diseases [242].
- *5-methoxytryptophan*. 5-methoxytryptophan, also called cytoguardin, is synthesized by 5-hydroxytryptophan methylation in fibroblasts and endothelial cells. It inhibits cyclooxygenase-2 (COX-2) transcription, an enzyme involved in the conversion of arachidonic acid to various prostaglandins, induced by diverse proinflammatory and mitogenic factors. Cytoguardin has been shown to defend against inflammation-mediated tissue damage and fibrosis. In contrast to serotonin, cytoguardin exerts anticancer effects and has the potential to be a therapeutic agent for certain types of cancer [219,220].
- *TRPH inhibitors*. The suppression of serotonin synthesis by administering TRPH inhibitors is promising in the treatment of several diseases, including cancer, gastrointestinal disorders, metabolic syndrome, NAFLD, fibrotic diseases, and cardiovascular diseases [50,51,145,160]. The investigation is focused on inhibitors that decrease serotonin synthesis but cannot cross the BBB. The first TRPH inhibitor approved by the FDA for therapy of diarrhea, cutaneous flushing, and bronchoconstriction due to carcinoid syndrome has been teloristat ethyl [62].
- *Selective serotonin reuptake inhibitors (SSRIs)*. SSRIs, such as fluvoxamine, sertraline, and citalopram, increase the concentration of serotonin in nerve synapses and are recognized as primary antidepressant drugs [239,243]. Controversial data exist regarding the use of SSRIs in cancer therapy [62]. The use of SSRI is associated with increased risk of bleeding, especially intracranially and in the upper gastrointestinal tract. The probable cause is decreased uptake of serotonin by thrombocytes from plasma, leading to impaired function [243].
- *Tetrahydrobiopterin (THB*). THBs are enzymatic cofactors required for the hydroxylation of AAA, including TRP, and NO synthesis. THB exerts antioxidant and anti-inflammatory effects and has been suggested as a candidate drug for the therapy of cognitively impaired patients experiencing metabolic disorders and nervous system diseases, including Alzheimer’s disease [60,244].
- 5-*HT receptor ligands*. Several agonists and antagonists of selective 5-HT receptors have been developed and clinically relevant drugs used or investigated for the therapy of IBD, schizophrenia, depression, migraine, obesity, cancer, and other diseases [148,149]. Antagonists of 5-HT_3_ receptors are used as antiemetics following chemotherapy [245].

### 11.2. Targeting the TRP-KYN Pathway

The dysregulation of the TRP-KYN pathway is implicated in a wide range of diseases. Whereas decreased flux through the TRP-KYN pathway plays a role in the pathogenesis of pellagra (Section 9.1), the TDO and IDO1 are activated, and the flux through the TRP-KYN pathway markedly increases in metabolic syndrome, cancer, and nervous diseases (Section 9.5, Section 9.6 and Section 9.9). A suggested marker of TRP-KYN pathway activity outside the liver is the plasma concentration and urinary excretion of QA, which accumulates in the body in a broad spectrum of diseases [246]. The cause is limited conversion of QA to nicotinic acid mononucleotide due to low QPRT activity in extrahepatic tissues. Strategies targeting the TRP-KYN pathway should also rebalance the levels of specific metabolites, primarily KYNA and QA, particularly in nervous system diseases. Achieving good BBB permeability remains a limitation for most TRP-KYN pathway inhibitors examined for the treatment of nervous diseases. Promising lines of investigation include:

- *TDO and IDO1 inhibitors*. Several dual (TDO/IDO1) inhibitors have been developed for cancer therapy and have entered clinical trials [222,225,247,248]. High expression of TDO in various forms of human cancer, especially bladder carcinoma, hepatocarcinoma, and melanoma, resulted in the investigation of antitumour properties of specific TDO inhibitors, such as taxifolin [249]. The therapeutic effect of specific IDO1 inhibitors, such as epacadostat and indoximod, appears to be less significant than that of dual inhibitors [248]. More perspective than enzyme inhibition is probably vaccination directed against IDO1-expressing cells [222].
- *KYNA and neuroprotective KYN derivatives*. The antioxidant and neuroprotective properties of KYNA (Section 7.2.3) indicate that it could be used in the therapy of neurodegenerative diseases [104,107,109,110]. Data from rodent studies indicate the benefits of KYNA in disorders associated with metabolic syndrome, including its effects on blood pressure and lipid metabolism [104,250]. Unfortunately, the studies examining the therapeutic potential of KYNA in humans are not available. KYNA is present in various kinds of food, and small amounts of KYNA of exogenous origin are present in the digestive system and circulation [250].
  The examples of KYN derivatives with neuroprotective and immunomodulatory effects include Laquinimod and Tranilast. Laquinimod (quinoline-3-carboxamide), probably via AHR activation in astrocytes, down-regulates migration of leukocytes, reduces inflammation and neuroaxonal damage, and is used for the treatment of multiple sclerosis [251,252]. Transilat, an anti-allergic agent investigated in a wide range of disorders, is a derivative of ANA [253].
- *KYN transaminase inhibitors*. The KYN transaminase inhibitors block the conversion of KYN to KYNA and are being investigated in the treatment of schizophrenia [254].
- *Kynurenine 3-monooxygenase inhibitors*. Inhibitors of kynurenine 3-monooxygenase limit the production of neurotoxic kynurenines and are being investigated for the treatment of spinal cord injury and neurological diseases, such as Parkinson’s, Huntington’s, and Alzheimer’s [255].
- *Suppression of QA formation*. Injections of 4-chloro-3-hydroxyanthranilate, a 3-HANA oxygenase inhibitor blocking the conversion of 3-HANA into QA, significantly improved functional recovery and preserved white matter in adult guinea pigs after spinal cord injury [256].
- *Chromium picolinate*. The chelator properties of PA are used to administer chromium for the treatment of chromium deficiency in individuals with diabetes and those undergoing weight reduction [257,258].
- *Kynurenines and transplantation*. Kynurenines with immunosuppressive effects, such as KYN, PA, 3-HKYN, and 3-HANA, are believed to suppress the T-cell response and promote tolerance to transplanted tissue [102,259].

### 11.3. Targeting TRP-IND Pathways

Indole and most of its derivatives, formed by gut microbiota, are potentially beneficial substances that, through their antioxidative properties and modulation of AHR and PXR, play a crucial role in gut barrier integrity, immune system function, and the gut–brain axis. Therefore, optimal composition of the intestinal microbiome is pivotal in maintaining homeostasis and health. The dysbiosis is implicated in aging and mood disorders, and the pathogenesis of various diseases, including metabolic syndrome, IBD, immune and neurological diseases, and cancer [5,260]. On the other hand, a detrimental effect may be an increased level of some indole derivatives in the blood, such as indoxyl sulfate and IAA, as occurs in uremia [195]. Therapeutic possibilities of targeting TRP-IND pathways include:

- *IPA*. The beneficial effects of IPA on maintaining intestinal barrier integrity, as well as its antioxidant, anti-inflammatory, and neuroprotective properties, suggest its potential use in the therapy of various diseases [115,132,133,168]. In animal and in vitro studies, IPA has been shown to exert cytostatic effects in cancer and to alleviate rheumatoid arthritis, steatohepatitis, and muscle protein breakdown in inflammatory states [135,226,261,262].
- *IAA*. The cytotoxic properties of IAA oxidation products led to the hypothesis that they could be used in cancer therapy. The anticancer properties of IAA coupled with horseradish peroxidase have been demonstrated under in vitro conditions [121,263]. Administration of IAA prevents bacterial translocation into the portal blood and protects against alcoholic steatohepatitis in mice [4].
- *Tryptamine*. Due to the ability of tryptamine to activate 5-HT and trace amine-associated receptors, several drugs derived from tryptamine have been developed to treat migraines and neuropsychiatric disorders [264].
- *Probiotics*. Probiotics are microorganisms that, when administered, bring beneficial health effects to the host. For example, administering *Lactobacillus* and *Bifidobacterium*, which produce AHR agonists, mitigates the detrimental effects of certain microbiota on gut barrier integrity and CNS function [6]. Probiotics have been used for the prevention of age-related disorders and the treatment of various diseases, including IBD, neurological disorders, metabolic syndrome, and liver cirrhosis [136,137,138].
- *Indole-3-carbinol*. It is a naturally occurring indole derivative found in cruciferous vegetables and a known ligand for AHR. Experimental studies have found that it can prevent colitis-associated microbial dysbiosis, repress colonic inflammation, and prevent hepatotoxicity, neuronal damage, and carcinogenesis induced by various chemicals [227,228].
- *AST-120*. A substance that reduces indole absorption from the gut and indoxyl-sulfate levels in plasma has been investigated in the therapy of CRI [196].

## 12. Summary and Conclusions

TRP is an essential amino acid that, through its role in the synthesis of proteins, serotonin, melatonin, nicotinamide nucleotides, and various KYN and indole derivatives, plays numerous biologically exceptional roles. Alterations in TRP metabolism have been demonstrated in many diseases, including those not mentioned in this review. Clinicians can use plasma concentrations and urinary excretion of TRP and its metabolites to assess disease progression and risk.

In the previous sections of this review, various therapeutic perspectives were shown by influencing individual pathways of TRP metabolism. Unfortunately, the understanding and characterization of the mechanism of action of most TRP metabolites are in their early stages. In most diseases, we do not know whether the given alterations in TRP metabolism contribute to the disease’s pathogenesis or are merely a consequence. The discrepancies and gaps also exist in the current understanding of the relationships and interactions among the three TRP metabolic pathways. For instance, TRP and most derivatives of its three metabolic pathways act as AHR ligands, and several studies have shown that changes in flux through the TRP-KYN pathway affect brain serotonin production. Furthermore, the effects and potential therapeutic uses of many TRP metabolites have not been fully explored. For example, the literature shows very limited knowledge about the effects of cinnabaric and picolinic acid. For targeting TRP metabolic pathways in the therapy of nervous diseases, the ability of individual TRP metabolites to cross the BBB is important. Melatonin, 5-hydroxytryptophan, some derivatives of the TRP-KYN pathway (e.g., KYN, 3-HKYN, and ANA), and the TRP-IND pathway, particularly IPA, can cross the BBB. On the other hand, serotonin cannot cross the BBB, and QA, KYNA, and 3-HANA cross the BBB poorly. There is a growing interest in and search for indole-derived compounds with therapeutic potential that are safe for human use.

Regarding the clinical perspectives, it can be suggested:

- Clinical research should prioritize longitudinal and interventional studies to establish causal links between TRP intake, microbiota-derived metabolites, and host metabolism and neuroimmune responses.
- Large randomized clinical trials are needed to define long-term efficacy and clinically relevant outcomes of TRP administration and targeting TRP catabolism pathways.
- Rigorous evaluation of the safety and dose–response of TRP supplementation will support the development of personalized nutritional and therapeutic strategies.

In conclusion, all pathways of TRP catabolism are altered across a broad spectrum of human illnesses, and further investigation is needed to better understand the role of TRP metabolism in disease pathogenesis. Current knowledge is insufficient to provide guidelines for the use of TRP and drugs that affect its metabolic pathways to achieve therapeutic and avoid detrimental effects in most clinical conditions. The goal for clinical research is to explore options for TRP-targeted therapies and their integration into new therapeutic strategies.

## Data Availability

No new data were created or analyzed in this study. Data sharing is not applicable to this article.

## Abbreviations

AAA, aromatic amino acids; ACMS, 2-amino-3-carboxymuconate semialdehyde; ANA, anthranilic acid; ARH, aryl hydrocarbon receptor; BBB, blood–brain barrier; BCAA, branched-chain amino acids (valine, leucine, and isoleucine); CRI, chronic renal insufficiency; CSF, cerebrospinal fluid; DHBP, dihydrobiopterin; ECC, enterochromaffin cells; IAA, indole-3-acetate; IBD, inflammatory bowel disease; IDO, indoleamine 2,3-dioxygenase; IPA, indole-3-propionic acid; KYN, kynurenine; KYNA, kynurenic acid; LNAA, large neutral amino acids; MAO, monoamine oxidase; NAFLD, nonalcoholic fatty liver disease; NMM, nicotinic acid mononucleotide; PA, picolinic acid; PRPP, 5-phosphoribosyl-1-pyrophosphate; PXR, pregnane X receptor; QA, quinolinic acid; QPRT, quinolinic acid phosphoribosyl transferase; RNS, reactive nitrogen species; ROS, reactive oxygen species; SSRI, selective serotonin reuptake inhibitors; TDO, tryptophan-2,3-dioxygenase; THBP, tetrahydrobiopterin; TRPH, tryptophan hydroxylase; TRP, tryptophan; T2DM, type 2 diabetes mellitus; XA, xanthurenic acid; 3-HKYN, 3-hydroxy-kynurenine; 3-HANA, 3-hydroxy-anthranilic acid; 5-HIAA, 5-hydroxyindole-3-acetic acid; 5-HT, 5-hydroxytryptamine (serotonin).
